# Draft Genome Sequence of the Axenic Strain *Phormidesmis* *priestleyi* ULC007, a Cyanobacterium Isolated from Lake Bruehwiler (Larsemann Hills, Antarctica)

**DOI:** 10.1128/genomeA.01546-16

**Published:** 2017-02-16

**Authors:** Yannick Lara, Benoit Durieu, Luc Cornet, Olivier Verlaine, Rosmarie Rippka, Igor S. Pessi, Agnieszka Misztak, Bernard Joris, Emmanuelle J. Javaux, Denis Baurain, Annick Wilmotte

**Affiliations:** a InBioS—Centre for Protein Engineering, University of Liège, Liège, Belgium; b InBioS—PhytoSYSTEMS, Eukaryotic Phylogenomics, University of Liège, Liège, Belgium; c UR-Geology—Palaeo-Biogeology-Botany-Palynology, University of Liège, Liège, Belgium; d Institut Pasteur, Unité des Cyanobactéries, Centre National de la Recherche Scientifique (CNRS) Unité de Recherche Associée (URA) 2172, Paris, France; e BCCM/ULC, University of Liège, Liège, Belgium

## Abstract

*Phormidesmis priestleyi* ULC007 is an Antarctic freshwater cyanobacterium. Its draft genome is 5,684,389 bp long. It contains a total of 5,604 protein-encoding genes, of which 22.2% have no clear homologues in known genomes. To date, this draft genome is the first one ever determined for an axenic cyanobacterium from Antarctica.

## GENOME ANNOUNCEMENT

Cyanobacteria are key organisms of the aquatic and terrestrial Antarctic food webs. They are the dominant primary producers and generally play a major role in the first steps of the colonization of deglaciated habitats (1). Thin filamentous cyanobacteria of the orders *Oscillatoriales* and *Synechococcales* (2) are abundant in Antarctica and usually form conspicuous mats and crusts in freshwater and terrestrial ecosystems. According to SSU rRNA gene phylogenies, they are clustered in several lineages, for which detailed data about their taxonomy and potential traits (e.g., nitrogen fixation, salinity tolerance) remain scarce. Moreover, no genome of Antarctic cyanobacteria is currently available.

The strain *Phormidesmis priestleyi* ULC007 (3) was originally isolated from Lake Bruehwiler (4), a shallow freshwater lake of 1 ha (5). It is located in the Larsemann Hills, where air temperatures vary from −18°C during winter to 10°C during summer (6), underlining the high variability of the climatic conditions.

The purification protocol consisted of successive streakings on solidified BG11-based culture medium until obtaining an axenic culture (7). The genomic DNA of ULC007 was sequenced in two runs. The first run was performed on an Illumina HiSeq 2500 System (Illumina, Inc., San Diego, CA, USA), which generated 4,404,753 paired-end reads of 100 bp. The second run, performed using the Illumina MiSeq System (Illumina Inc, San Diego, CA, USA), generated 440,221 paired-end reads of 250 bp.

Raw reads from both runs were processed and *de novo* assembled using Velvet v1.2.10 (8) and SPAdes v3.5 (9) after a thorough analysis of the kmer length distribution using kmergenie v1.6982 (10). The resulting sets of contigs were integrated using the CISA v1.3 software (11). The accuracy of the final assembly was evaluated using the REAPR v1.0.18 software (12). Contigs were annotated using glimmer3 v3.02 (13) and a reference database composed of 260 cyanobacterial genomes extracted from the PATRIC database. The presence of secondary metabolite biosynthesis gene clusters was tested using antiSMASH v3.0 (14).

The draft genome of ULC007 is composed of 5,684,389 bp distributed into 118 contigs, with an average G+C-content of 48.6%. Final assembly yielded an *L*_50_ value of 12 (*L*_90_ = 51) and an *N*_50_ value of 152,457 bp (*N*_90_ = 27,655 bp). A total of 5,604 protein-encoding genes (PEGs) were identified by glimmer3, of which 4,785 PEGs correspond to proteins already characterized in other cyanobacterial genomes, whereas 1,244 PEGs are apparently new. The genome contains a variety of PEGs involved in stress response (e.g., molecular chaperones DnaK and DnaJ, DNA gyrase, putative cold shock proteins of the Csp-family). The main components (KaiA, KaiB, and KaiC) of the cyanobacterial circadian clock were found, as well as PEGs related to the circadian input (CikA, LdpA, Pex) and output (CikA, SasA, RpaA, LabA) pathways (15). Finally, three unknown NRPS-, NRPS/PKS-, and PKS-like gene clusters were identified, whose exploration may potentially lead to the discovery of novel secondary peptide metabolites.

This genome is the first Antarctic cyanobacterial genome ever determined, and undoubtedly will contribute to our understanding of the mechanisms underpinning the survival and ecological success of cyanobacteria in high latitudes.

### Accession number(s).

This whole-genome shotgun project has been deposited at DDBJ/ENA/GenBank under the accession MPPI00000000. The version described in this paper is version MPPI01000000.
